# Tetanus in a vaccinated soil expert - a case report

**DOI:** 10.1186/s12879-023-08780-1

**Published:** 2023-11-04

**Authors:** Olanrewaju Medu, Nazlee Ogunyemi, Maurice Hennink, Shawn Mawer, Theresa Styles-Mackinnon, Alex Wong, Charles Omosigho, Tania Diener

**Affiliations:** 1 Saskatchewan Health Authority, Population & Public Health, Regina, Canada; 2 Community Health and Epidemiology, University of Saskatchewan, Saskatoon, Canada; 3 Saskatchewan Health Authority, Population & Public Health, Weyburn, Canada; 4 Saskatchewan Health Authority, Regina General Hospital, Regina, Canada; 5 Saskatchewan Health Authority, St. Joseph’s Hospital, Estevan, Canada

**Keywords:** Tetanus, Immunization, Abrasion, Occupational health

## Abstract

**Background:**

Tetanus is a life-threatening vaccine-preventable disease found more commonly in tropical climates. It accounted for up to 60 deaths annually until the introduction of the tetanus toxoid. It is now rare in Canada by immunization. This study aims to describe a case of tetanus in Saskatchewan with possible occupational exposure.

**Case Report:**

We describe the case of a vaccinated soil expert with tetanus resulting from skin abrasions. This report highlights the early diagnosis, atypical presentation with possible vaccine attenuation and management approach, including acute care, intensive care unit referral, surgical management and physical rehabilitation. We also describe the public health management provided in this case. Despite the severity, the patient returned to his previous home environment with minimal motor sequelae.

**Conclusion:**

Occupational exposure to tetanus is an important risk, even in regions where the disease is rare. Prevention through vaccination is a key public health intervention that must be encouraged to avoid health complications that are often fatal.

## Introduction

Tetanus is a life-threatening but preventable disease with a poor outcome in the absence of early treatment [1]. It is caused by a neurotoxin of the bacterium *Clostridium tetani*, whose spores are widely distributed in soil worldwide [2]. Tetanus is found more frequently in tropical climates and accounted for almost 60,000 deaths worldwide in 2015 [1, 3]. The bacterium creates two toxins, tetanolysin and tetanospasmin. Although the function of tetanolysin is not yet understood, tetanospasmin leads to the disinhibition of the motor and autonomic systems, resulting in uncontrolled muscle contractions [4].

Tetanus is currently rare in Canada. In the 1920s and 1930’, between 25 and 60 deaths were reported annually [2]. However, with the introduction of tetanus toxoid in 1940, morbidity and mortality rapidly declined [5]. Recent Canadian data from 1990 to 2010 shows an average of four cases reported annually. During this period, persons 60 years of age or greater accounted for 48% of the cases [2].

## Case History

A 33-year-old Canadian-born man presented to an emergency department in Saskatchewan in October 2017 with a 90-minute history of lower back spasms and lumbar pain. Two days before presentation, he reported getting sick with nausea and vomiting followed by intense spasms in the back and limbs. Ten days before the onset of symptoms, he suffered an injury to his hands following involvement in a physical altercation, resulting in small cuts and abrasions on his hands, knuckles and fingers. He did not seek medical attention for these injuries.

His medical history included a history of involvement in a motor vehicle accident ten years prior and asthma. Medication history was not significant, with no history of chronic medication. He had previously received at least five tetanus-containing vaccines, with none in adulthood and the last dose about twenty years prior.

He resided in Saskatchewan and lived and worked across three prairie provinces as a soil testing technologist, where he was regularly exposed to dirt and soil.

On examination at the local hospital Emergency Department, he was observed to have an opisthotonic posture with spontaneous tetanic spasms. The spasms were described as intense, with associated grunts and diaphoresis. The patient stayed conscious during the spasms. Back spasms progressed to arching of the back with continued spasms of the arms and legs. The spasms were also associated with certain triggers, including noise and movement. Involvement of the jaw muscles was not observed.

He was commenced on antibiotics, antispasmodic agents and pain relief, including opioids and benzodiazepines. However, due to worsening hypercapnic respiratory failure, likely due to benzodiazepines, he was transferred to a larger regional facility.

At the regional hospital, the patient continued to manifest opisthotonic posturing while having spasms, with autonomic symptoms including diaphoresis and drenching the examination bed. Opisthotonus could be elicited with mild palpation of the paraspinal muscles. No tightening of facial muscles (*risus sardonicus*) nor signs of meningism were present clinically. He was admitted to the intensive care unit (ICU) and intubated. A clinical diagnosis of generalized tetanus was made based on the history, physical findings and laboratory investigations that excluded other differential diagnoses.

Clinical management involves ongoing supportive therapy and adequate pain management. A single dose of 500 units of tetanus immunoglobulin was administered intramuscularly for the neutralization of unbound tetanus toxin. No wound infiltration was done as there was no visible wound at presentation. While on admission, the patient was commenced on a three-dose series of tetanus-diphtheria-acellular pertussis (Tdap) vaccine consistent with treatment guidelines [6].

The patient spent 17 days in hospital following the acute episode, including one day in the local hospital and another 16 in the regional hospital. Public health follow-up 13 days post-discharge revealed that he had ongoing muscle spasms and spasticity in addition to difficulty ambulating, requiring a walking aid. He was found to have musculoskeletal complications from the tetanus disease resulting in a slipped disc for which he required operative repair with post-operative physiotherapy. He was referred to the neurosurgery and physical medicine teams for additional clinical management. Two years post-incident, he has since returned to work without further physiotherapy follow-up planned.

## Discussion

This case report highlights the importance of up-to-date tetanus immunization, clinical features of generalized tetanus, management challenges experienced during this patient’s clinical course, and inconsistent approaches around appropriate dosing of tetanus immunoglobulin.

Tetanus is a vaccine-preventable disease caused by *Clostridium tetani*, a spore-forming anaerobic bacterium found commonly in soil or in the gastrointestinal tracts of mammals, which produces a potent neurotoxin, tetanospasmin [7]. The usual incubation ranges from 3 days to 3 weeks [7]. However, it has been reported that the incubation period may vary depending on distance from the injury site to the central nervous system [8].

The neurotoxin tetanospasmin causes violent spastic paralysis by blocking the release of γ-aminobutyric acid, an inhibitory neurotransmitter acting on motor neurones producing muscular rigidity by raising the resting firing rate of motor neurons and generating spasms by modifying reflex responses to afferent stimuli [7, 9].

Tetanus typically follows a recognized injury and may be associated with contamination of wounds with soil, manure, or rusty metal [10]. It is important to note, however, that injuries may be trivial, and in 15–25% of patients there is no evidence of a recent wound [10]. Other risk factors include dental procedures, surgery, intramuscular injections, intravenous drug use and burns [10].

There are several clinical manifestations of tetanus, including neonatal, cephalic, localized and generalized tetanus [11]. The manifestations of tetanus based on this classification reflect host factor differences and the site of inoculation.

Generalized tetanus is the most common form accounting for more than 80% of cases [12]. It typically presents in a descending pattern, with trismus followed by neck stiffness, difficulty swallowing, and abdominal muscle rigidity. Spasms may occur frequently, and complete recovery may take months [12]. The localized form of the disease is unusual. It produces painful spasms close to the wound site [12]. It can result in progression to generalized tetanus but is typically milder [12]. Cephalic tetanus is the rarest form and occurs when localized tetanus from a head wound affects the cranial nerves, leading to a predominant pattern at the presentation of paralysis rather than spasm; progression to generalized tetanus is common [7, 10]. Neonatal tetanus causes over 50% of tetanus-related deaths worldwide and is caused by poor umbilical hygiene [10]. Presentation occurs within one week of birth and involves failure to feed, vomiting, and generalized spasms [10]. Neonatal tetanus is rare in developed countries and entirely preventable by maternal vaccination [10]. Of note, *risus sardonicus* was not identified in this case and the reasons for this are unclear. It may have been due to non recognition in the early stages of an atypical presentation [13, 14].

The development of tetanus in this particular case was due to the unlikely convergence of factors namely, waning immunity to tetanus due to not being up to date with tetanus containing vaccines per guidelines; ongoing workplace exposures due to work as a soil scientist and the hand abrasions following the altercation which provided a path for the bacteria to be inoculated.

Tetanus is a clinical diagnosis primarily based on relevant history, a physical examination of the patient and excluding other differential diagnoses. Currently, there are no laboratory tests that can confirm tetanus.

Tetanus management principles include initiating supportive therapy, where appropriate, debriding the wound to eradicate spores, altering germination conditions where applicable, and stopping the production of toxins within the wound [10, 15]. This is primarily achieved through antibiotic therapy [10, 15]. However, as there was no wound to be treated, this was not required in our case.

Additional measures include neutralizing unbound toxins. The use of tetanus immune globulin (TIG) is recommended for treating tetanus to remove further fixation of neurotoxins in the CNS [7]. Deciding on the appropriate dosing of TIG was challenging as there were two slightly different management options. Previously recommended guidance suggested wound infiltration with larger TIG doses in the range of 3,000–6,000 units [7, 11], while more recent evidence suggests a single dose of 500 units may be equally effective [9, 16].

A major component of disease management and rehabilitation involves managing manifestations such as rigidity and tetanic spasms and other complications such as laryngospasms, fractures, hypertension, nosocomial infections, pulmonary embolism, and aspiration pneumonia [17].

Furthermore, as tetanus disease does not result in lifelong tetanus immunity, active immunization with a total of three doses of tetanus and diphtheria toxoid spaced at least two weeks apart with a tetanus toxoid-containing vaccine is advised and preferably early in the course of recovery [6].

## Conclusion

This case report emphasizes some important features in diagnosing and managing tetanus. First, we can highlight that abrasions are important exposures to consider in addition to puncture wounds and other previously documented routes of infection, such as dental procedures, surgery, intramuscular injections, intravenous drug use and burns. We also highlighted the importance of regular tetanus boosters during adulthood, consistent with national guidelines. We conclude that if the patient had received a dose of tetanus-containing vaccine in adulthood, specifically in the last ten years or if he had accessed emergency room care and received a tetanus booster, he likely may have had better protection, and tetanus would not have occurred in this patient. An important consideration is that for occupations that work with soil and other media that may expose them to *Clostridium tetani*, regular booster doses in adulthood and post-exposure should be considered an occupational requirement. Finally, we highlight the need for consistent directions on the appropriate dosing of tetanus immunoglobulin.

## Data Availability

The data used during this study is available from the corresponding author on reasonable request.
